# Rhabdomyosarcoma and pleomorphic sarcoma in the same location: Recurrence or new entity?

**DOI:** 10.1007/s00508-021-01872-5

**Published:** 2021-04-27

**Authors:** Daniel Steiner, Maria Anna Smolle, Iva Brcic, Andreas Leithner

**Affiliations:** 1 Department of Orthopedics and Trauma, Medical University of Graz, Auenbruggerplatz 5, 8036 Graz, Austria; 2 Diagnostic and Research Institute of Pathology, Medical University of Graz, Neue Stiftingstalstraße 6, Graz, 8010 Austria

**Keywords:** Soft tissue sarcoma, Radiotherapy, Radiation-associated soft tissue sarcoma, Sarcoma pathology, Recurrence

## Abstract

Soft tissue sarcomas (STS) represent a small group of adult solid malignancies, with risk factors such as environmental factors, genetic predisposition, and prior radiotherapy. In STS patients with a novel swelling, differential diagnoses include recurrence, second primary cancer, metastasis from unknown primary cancer, and radiation-associated STS, the latter usually occurring approximately 10 years after radiotherapy. We present the case of a 64-year-old male patient with pleomorphic rhabdomyosarcoma, who underwent resection and radiotherapy. The patient presented again 5 years later with painful swelling in the area of the prior sarcoma, raising suspicion of recurrence. Resection was performed and a diagnosis of pleomorphic sarcoma (not otherwise specified [NOS]) was made. The patient was treated with radiotherapy and remained sarcoma-free for the following 7 years. A molecular analysis of both neoplasms, using RNA next-generation sequencing, did not detect any specific fusions. Due to the lack of rhabdomyoblastic differentiation in the second sarcoma and the low likelihood of a second primary in the same previously irradiated location, the diagnosis of a radiation-associated STS was suggested. This short report illustrates the difficult diagnostic work-up of a presumably radiation-associated STS, as these neoplasms lack characteristic morphological and immunohistochemical features. In our case, the suggested diagnosis may have pointed against another course of radiotherapy in an already irradiation-harmed region. Therefore, a relatively low latency period between surgery, radiotherapy, and diagnosis of another STS should not automatically point towards recurrence and may prompt further in-depth investigation.

## Introduction

Sarcomas in general account for less than 1% of all adult solid malignancies and of all sarcomas the vast majority are soft tissue sarcomas (STS) with an estimated incidence of 3–4 per 100,000 patients per year [1]. While the risk factors for developing STS are still poorly understood, it seems that environmental factors, genetic predisposition, and interactions between these two play a substantial role. Furthermore, prior radiotherapy increases the risk of developing STS [2].

Due to the low incidence and more common differential diagnoses, STS are difficult to diagnose. Differential diagnoses in patients presenting with soft tissue swelling include preceding trauma with concurrent hematoma as well as a metastasis from unknown primary cancer. The STS patients presenting years after initial diagnosis with a novel swelling are highly suspicious for recurrence; however, second primaries or soft tissue metastasis from other cancers should be considered. Another differential diagnosis to keep in mind is radiation-associated STS, usually occurring approximately 10 years after radiotherapy [3, 4].

In the following short report, the history, management, and later diagnostic work-up of a patient with two STS in the same location, occurring over a prolonged period are presented.

**Fig. 1:**
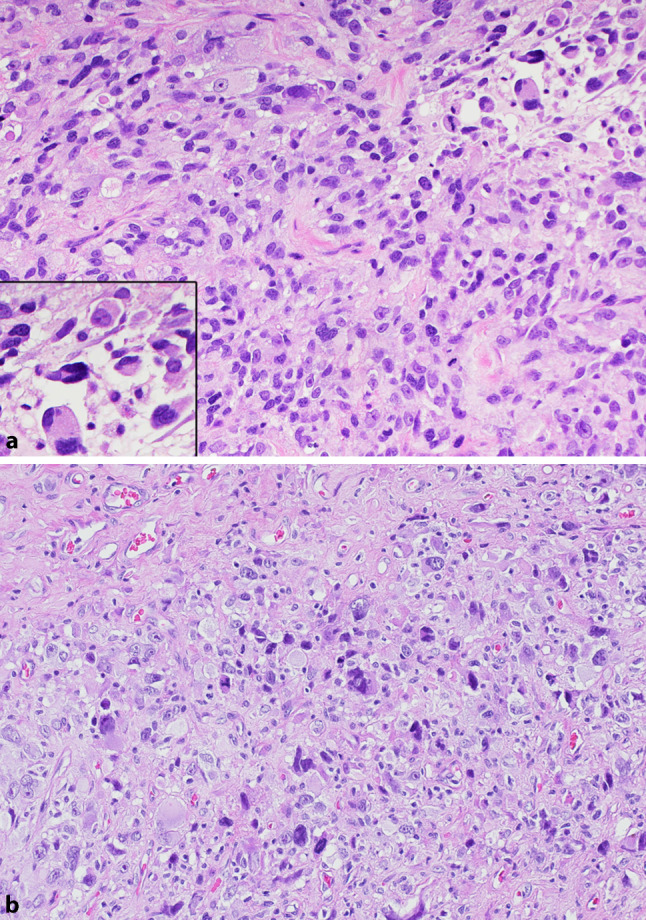
Histological analysis of the first lesion (HE × 20; panel **a**), showing a malignant mesenchymal neoplasm composed of haphazardly arranged spindle-shaped and pleomorphic cells with prominent nucleoli and prominent eosinophilic cytoplasm (inset). Histological analysis of the second lesion (HE × 20; panel **b**), showing a malignant mesenchymal neoplasm composed of pleomorphic cells with enlarged irregular nuclei, focally prominent nucleoli and abundant eosinophilic cytoplasm. Numerous mitotic figures are present

In 2008, a 64-year-old male patient presented to our outpatient clinic after a tumor excision located on his right forearm at a different hospital. Histological analysis revealed a malignant mesenchymal neoplasm composed of haphazardly arranged spindle-shaped and pleomorphic cells with prominent nucleoli and prominent eosinophilic cytoplasm (Fig. 1). Immunohistochemically, cells showed positive reaction for desmin, myo-FD5, and anti-smooth muscle actin (SMA). These findings rendered the diagnosis of pleomorphic rhabdomyosarcoma, G3. Re-resection was subsequently performed, resulting in a wide R0 resection. Computed tomography (CT) scans of the thorax, abdomen, and pelvis showed no evidence of metastasis. The split-skin graft of the forearm had to be revised 2 weeks after the resection due to a wound healing deficit. Postoperative management included radiotherapy of the right forearm with a dose of 50 Gy applied in 25 fractions, followed by a boost with 10 Gy in 5 fractions, as well as regular follow-ups. The patient remained sarcoma-free for the following 5 years.

During the patient’s stay at our center, a melanoma located in the neck was excised. The melanoma was not ulcerated and infiltrated 0.75 mm into the dermis (AJCC 2018: pT1a). Due to positive resection margins of the first excision, a re-resection with safety margins of 1cm was performed. Histological analysis revealed a melanocytic nevus without further evidence of melanoma.

In 2013, 5 years after the first sarcoma resection, the patient presented with painful swelling of his right forearm, which he had first noticed 1 month previously, without preceding trauma. Since 2008, multiple nevi and another melanoma, located in the thoracic region, had been excised, but the patient could not provide further information or medical records regarding this topic. Magnetic resonance imaging (MRI) showed a 2 cm large expansion in the subcutis arising at the same location as the pleomorphic rhabdomyosarcoma resected in 2008. The lesion showed contrast enhancement and was directly attached to the muscular fascia. These findings suggested recurrence.

An incisional biopsy was performed and showed a malignant mesenchymal neoplasm composed of pleomorphic cells with enlarged irregular nuclei, focally prominent nucleoli, and abundant eosinophilic cytoplasm. Numerous mitotic figures were present (Fig. 1). Immunohistochemically, cells were negative for desmin, myo-FD5, SMA, S100, melan A, and HMB45. The diagnosis of pleomorphic sarcoma (NOS), occurring in the same location as the pleomorphic rhabdomyosarcoma 5 years before, was made. Although biopsy material did not show rhabdomyoblastic differentiation, recurrence was suggested as the most likely diagnosis.

A wide resection was performed but histological analysis revealed positive resection margins. A subsequent re-resection resulted in negative margins. Negative pressure wound therapy and split-skin graft were subsequently applied to support wound healing. Subsequently, the patient underwent radiotherapy of the right forearm, with a dose of 25 Gy applied in 25 fractions. He remained disease-free for the following 7 years up to this date with regular follow-ups consisting of thoracic CT scans and MRI scans of the right forearm.

In 2020, the molecular analysis (Archer® FusionPlex Sarcoma panel; ArcherDX, Inc., Boulder, CO, US) using next-generation sequencing-based anchored multiplex PCR technique, which had not been available at our institution when the patient underwent treatment, was performed on both the pleomorphic rhabdomyosarcoma and the pleomorphic sarcoma (NOS). The panel searched for fusion in the following genes: *ALK, BCOR, BRAF, CAMTA1, CCNB3, CHMP2a, CIC, EPC1, EWSR1, FOS, FOSB, FOXO1, FUS, GLI1, HMGA2, JAZF1, KMT2a, MEAF6, MGEA5, MKL2, NCOA2, NTRK1, NTRK2, NTRK3, PAX3, PDGFB, PLAG1, RAB7a, RET, ROS1, SS18 (SYT), STAT6, TAF15, TCF12, TFE3, TFG, TGFBR3, USP6, VCP, and YWHAE.* In both specimens, no specific fusions were detected. A pathological re-evaluation confirmed the diagnoses of a pleomorphic rhabdomyosarcoma and a pleomorphic sarcoma (NOS). Due to the low likelihood of a second primary in the same previously irradiated location, the diagnosis of a radiation-associated STS was suggested.

## Discussion

In the current case a patient presented with painful swelling of his right forearm and history of pleomorphic rhabdomyosarcoma with subsequent radiotherapy in the same location as well as two melanomas located elsewhere. Pathological analysis of the specimen showed pleomorphic sarcoma (NOS). Although neither histomorphological nor immunohistochemical analysis proved it to be a recurrence of the known pleomorphic rhabdomyosarcoma or a metastasis of the melanoma, recurrence was the most likely differential diagnosis. This assumption was supported by the short latency period of 5 years between radiotherapy and diagnosis of pleomorphic sarcoma (NOS), together with the low probability of a different sarcoma in the same location. The postulated recurrence was treated with wide resection and radiotherapy.

A fusion panel, performed on both neoplasms (primary specimen and suspected recurrence) to gain more knowledge of the neoplasm’s biology, showed no specific fusions in both specimens. The immunohistochemical evaluation confirmed the lack of rhabdomyoblastic components in the second neoplasm, thus arguing against local recurrence but favoring the second neoplasm. Due to preceding radiotherapy exposure, it was concluded that the STS may have been triggered by radiotherapy of the first sarcoma, thus possibly constituting a radiation-associated STS.

According to the literature the median time between radiotherapy and the occurrence of radiation-associated STS ranges between 9.3 years [3] and 10 years [4], with some cases reported occurring as early as 1.2 years after surgery [3]. In our case, the latency period was 5 years, which is considerably below the median time range. Criteria to differentiate between a radiation-associated and a sporadic sarcoma were suggested by Cahan in 1948 [5], including a latency period of over 5 years; however, in 1971, Arlen et al. described time frames starting with 4 years [6]. Therefore, lower latency periods than the median time of about 10 years mentioned above must be expected.

The problem in our case was the short latency period between radiotherapy and the occurrence of the second neoplasm, pointing away from radiation-associated STS, and the relatively obvious differential diagnosis of a local recurrence due to the location of the second neoplasm; however, considering that radiation-associated STS can occur with a latency period considerably below the median time range reported in the literature and that the second neoplasm did not show rhabdomyoblastic differentiation, radiation-associated STS rather than local recurrence seems reasonable.

The differentiation between a recurrent and a secondary radiation-associated malignancy is crucial for patients’ treatment. The subsequent radiotherapy in a possibly radiation-associated STS may have been avoided in this patient. Furthermore, radiation-associated sarcomas have worse disease-specific survival than sporadic sarcomas [3, 4], as well as a higher local recurrence risk [3], suggesting more thorough clinical follow-up. Up to today, our patient is free of disease, despite having undergone further radiotherapy of the presumably radiation-induced STS. Yet, reaching the suggested diagnosis years earlier by combining history, examination, radiology, and histopathology, may have spared the patient from additional irradiation.

Limitations of our diagnostic work-up include the absence of typical morphology and lack of robust markers for the diagnosis of radiation-associated STS. Prieto-Granada et al. reported the usefulness of loss of H3K27me3 expression as a reliable ancillary marker in the context of radiation-associated or sporadic malignant peripheral nerve sheath neoplasm [7]. Unfortunately, no studies on radiation-induced pleomorphic sarcomas have been reported.

This case illustrates the difficult diagnostic work-up of a presumably radiation-associated STS, as these neoplasms lack characteristic morphological and immunohistochemical features. In our case, this diagnosis may have pointed against another course of radiotherapy in an already irradiation-harmed region. Therefore, a thorough medical history with examination and radiology should be combined with histopathology for a diagnosis that is as accurate as possible. Furthermore, a relatively low latency period between surgery, radiotherapy, and diagnosis of another STS should not automatically point towards recurrence and may prompt further in-depth investigation.
